# Breaking Barriers in Orthodontics: An Experimental Study on How Stabilization Discs Improve Mini-Implant Outcomes

**DOI:** 10.3390/dj13030109

**Published:** 2025-02-28

**Authors:** Tinela Panaite, Cristian Liviu Romanec, Mihnea Iacob, Carina Balcos, Carmen Savin, Nicolae Daniel Olteanu, Raluca-Maria Vieriu, Chehab Alice, Irina Nicoleta Zetu

**Affiliations:** Department of Oral and Maxillofacial Surgery, Faculty of Dental Medicine, “Grigore T. Popa” University of Medicine and Pharmacy, 16 Universitatii Str., 700115 Iasi, Romania; tinela-panaite@umfiasi.ro (T.P.); mihnea.iacob@umfiasi.ro (M.I.); carina.balcos@umfiasi.ro (C.B.); carmen.savin@umfiasi.ro (C.S.); daniel.olteanu@umfiasi.ro (N.D.O.); raluca-maria.vieriu@umfiasi.ro (R.-M.V.); alice-chehab@umfiasi.ro (C.A.); irina.zetu@umfiasi.ro (I.N.Z.)

**Keywords:** finite element analysis, mini-implant orthodontic, orthodontic treatment, stabilization disk, stress distribution

## Abstract

**Background/Objectives:** The stabilization disc (SD) for orthodontic mini-implants is a novel device designed to enhance anchorage stability and minimize the risk of mini-implant mobility. The disc features a flat structure with four prongs and is crafted from biocompatible materials such as titanium or stainless steel. It provides additional support to mini-implants by improving force distribution and reducing stress concentration around the insertion site. This study aims to evaluate the biomechanical performance of mini-implants with an SD compared to without-SD mini-implants, with a specific focus on their ability to maintain anchorage under orthodontic loading conditions. **Methods**: A finite element analysis (FEA) model was created for a commercially available mini-implant (2.0 mm in diameter and 12 mm in length). The mandible’s anatomical structure was reconstructed in 3D from computed tomography (CT) scans using SpaceClaim software 2023.1. To simulate real-world orthodontic conditions, forces of 10 N were applied at an angle of 30°. This retrospective study explores the role of SDs in enhancing mini-implant stability by reducing displacement and optimizing stress distribution. The evaluation included analyzing von Mises stress, cortical bone deformation, and mini-implant movement under simulated orthodontic loading. **Results***:* The results demonstrate that the SD significantly reduces maximum total displacements by over 41% and redistributes von Mises stresses more evenly across the mini-implant and surrounding bone. Cortical bone stress and deformation were reduced in cases utilizing the SD, indicating enhanced implant stability and durability. **Conclusions**: The stabilization disc enhances mini-implant stability by improving stress distribution and reducing deformation without requiring permanent implant modifications. Its adaptability makes it a valuable solution for managing variable bone density and high orthodontic forces, offering a promising advancement in orthodontic anchorage.

## 1. Introduction

Finite element analysis (FEA) is a valuable tool in orthodontics, enabling precise modeling and analysis of the mechanical behavior of mini-implants under various loading conditions. By simulating stress distribution, placement angles, and surface modifications, FEA helps optimize biomechanical forces, enhance mini-implant stability, and minimize treatment complications [1,2,3]. This approach improves the success rate of orthodontic treatments, providing better outcomes and patient satisfaction.

Orthodontic mini-implants play an important role in providing stable anchorage during treatment, and their mechanical behavior under different conditions has been extensively studied. Sivamurthy and Sundari [1] examined stress distribution at mini-implant sites, emphasizing the importance of implant dimensions and loading directions in computational modeling. Similarly, Allum et al. [2] compared various implant sizes, demonstrating how differences in loading conditions influence bone response. Rito-Macedo et al. [3] further highlighted the importance of assessing peri-implant bone stress to optimize implant design.

The primary stability of the mini-implant orthodontic and the optimization of stress distribution are critical factors that contribute to reducing failure rates. Primary stability is primarily determined by the mechanical engagement of the mini-implant with the surrounding bone, which is influenced by several factors including the quality of the bone, the design of the implant, and the insertion technique [4,5]. Studies indicate that adequate primary stability is essential for the immediate loading of mini-implants, as any loosening can lead to treatment failure.

The mechanical properties of mini-implants, including their design and surface characteristics, play a significant role in stress distribution during orthodontic loading. Research has shown that the stress distribution around mini-implants can be optimized by considering factors such as the angle of insertion and the thickness of the cortical bone at the placement site [6,7]. FEA have demonstrated that mini-implants placed in optimal locations experience less stress and displacement under orthodontic forces, which correlates with higher success rates [7,8]. Furthermore, the use of surface treatments on mini-implants has been shown to enhance their stability and resistance to failure by improving the mechanical retention between the implant and the bone [5,9].

The success rates of mini-implants can vary significantly based on the magnitude and direction of the applied forces, as well as the operator’s experience and technique [10,11]. Studies have reported failure rates ranging from 5% to 28%, emphasizing the importance of careful planning and execution in mini-implant placement [10].

Conversely, clinical investigations, such as those conducted by Shahanamol et al. [12], present tangible benefits of mini-implants, including immediate loading, versatility in placement sites, and simplified procedures. These studies complement mathematical analyses by offering empirical validation from real-world scenarios and taking into account factors such as patient comfort and cost effectiveness [12]. Clinical studies emphasize the significance of considering hard and soft tissue interactions at implant sites, which mathematical models may not fully capture [13]. To enhance the performance and durability of orthodontic mini-implants, integrating mathematical analysis into the design process is essential. Katić et al. [14] focus on the geometrical design characteristics of mini-implants and their impact on insertion torque, highlighting the need to consider these factors for optimal performance. Additionally, Redžepagić-Vražalica et al. [15] compare different mini-implant designs and stress the significance of primary stability in enhancing performance. Moreover, computational biomechanics, as discussed, can aid in optimizing implant designs by considering factors like durability and kinematics simultaneously [16]. Wu et al. [8] demonstrate the optimization of the thread height and pitch of mini-implants to improve osseointegration and reduce stress levels in the surrounding bone. Furthermore, finite element analysis (FEA) plays an important role in assessing stress distribution and stability, as shown by Choi et al. [17] in their evaluation of stress on cortical bone post-loading. Future research directions in mini-implant orthodontics involve exploring stress distribution patterns at mini-implant sites during orthodontic movements, such as retraction, intrusion, and molar intrusion [1]. Furthermore, mathematical analysis can be utilized to assess the primary stability of mini-implants with different materials, contributing to advancements in anchorage techniques [18].

The incorporation of stabilization discs (SDs) in mini-implant systems enhances primary stability and reduces failure rates by optimizing stress distribution and mechanical resistance. This study hypothesizes that mini-implants with an SD will demonstrate superior biomechanical performance compared to those without an SD, particularly in maintaining anchorage under orthodontic loading conditions. 

## 2. Materials and Methods

### 2.1. Ethical Clearance

This study received ethical approval from the Research Ethics Committee of Grigore T. Popa University of Medicine and Pharmacy Iasi (Approval No. 178/02.05.2022).

### 2.2. Patent—Pending

The SD utilized in this study is a service invention currently under the process of patenting. It has been officially submitted for patent application under the title “Disc de Stabilizare pentru Mini-Implantul Ortodontic” (Stabilization Disc for Orthodontic Mini-Implants) with the application number 2024 00551, filed on 19 September 2024. The invention is set to be published in the Official Bulletin of Industrial Property—Inventions Section No. 2 of 2025, in compliance with applicable intellectual property laws. Detailed technical descriptions, claims, and designs related to the invention are available for public access at OSIM (State Office for Inventions and Trademarks) beginning 28 February 2025.

### 2.3. Geometric Modeling

**Figure 1:**
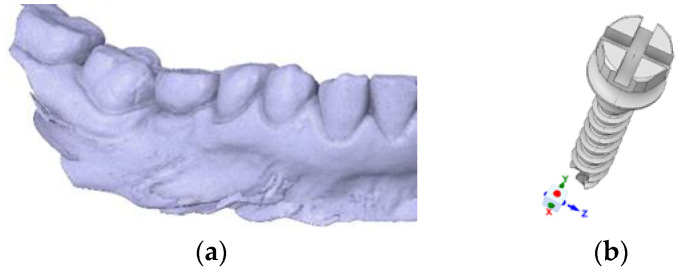
The finite element modeling methodology employed in this study: (**a**) mandibular scan image in STL format; (**b**) CT-scanned image in STL format of mini-implant model 1 and the resulting CAD model from modeling in Spaceclaim software.

The three-dimensional geometric models of the implantation bone sites and implants were constructed using Spaceclaim software (19.2, ANSYS, Inc., Canonsburg, PA, USA). The finite element analysis (FEA) was performed using ANSYS Workbench 19.2 (ANSYS, Inc., Canonsburg, PA, USA). The geometric model of the mandible was generated from an optical scan image saved in STL format, ensuring accurate anatomical representation. This model was further simplified geometrically to optimize computational stability and efficiency for finite element analysis (Figure 1a).

The mini-implant model used was designed based on commercial models from Dual Top Jeil Medical Corporation^®^ Seoul, Republic of Korea (Figure 1b). The material from which the analyzed implant system was manufactured was Ti6Al4V alloy. The dimensional parameters used for the implant system were as follows: length 10 mm/diameter 1.6 mm. A geometric model of the simulated implant system is presented in Figure 1b. The geometric model of the mini-implant was created using a scanned image in STL format, retaining all geometric dimensions for stress state, displacement, and deformation as close to reality as possible. These were investigated using the finite element modeling program Ansys. Material properties used in the finite element analysis were defined by their elastic modulus and Poisson’s ratio, as detailed in Table 1.

### 2.4. Structural Analysis

#### 2.4.1. Overview of the SD

**Figure 2:**
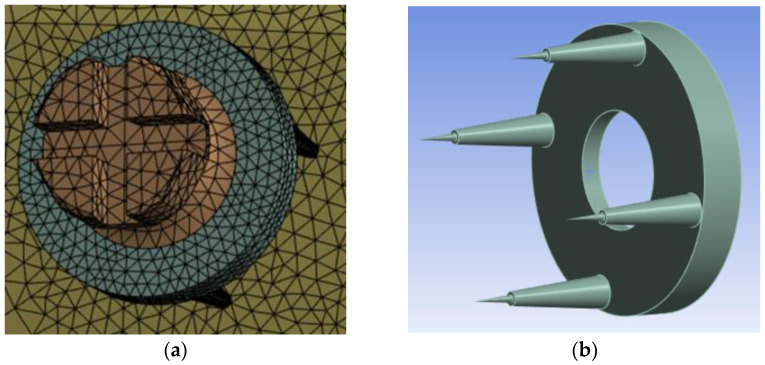
SD design: flat disc—the hole at the disc level has the same size as the neck of the mini-implant. The surface of the disc is smooth and made of the same material as the mini-implant—Ti6Al4V.

The structural static analysis was conducted assuming that the structure is an undamped system, disregarding any material behavior inhomogeneities. The stiffness was specified using the elastic and isotropic material model. The invention, known as the SD, is a medical device designed as an interchangeable disc to tackle problems related to the mobilization and instability of mini-implants utilized in orthodontic treatment (Figure 2a,b).

#### 2.4.2. Geometric Details of the Stabilization Disc (SD)

The stabilization disc (SD) has been meticulously engineered to ensure secure and precise placement within the bone structure, meeting the requirements of advanced orthodontic applications. The design incorporates specific dimensions tailored for its functionality, as follows.

##### General Dimensions

The ring diameter is 4 mm, with an internal diameter of 1.5 mm and an external diameter of 3 mm. These measurements ensure compatibility with standard mini-implant systems and promote mechanical stability during orthodontic procedures.

The leg height is set to 4 mm, providing sufficient penetration into the bone to enhance anchorage while minimizing unnecessary stress on the surrounding tissues.

The maximum insertion depth is limited to 1 mm to avoid excessive cortical bone perforation, ensuring precise and controlled placement during insertion.

##### Structural Design

**Figure 3:**
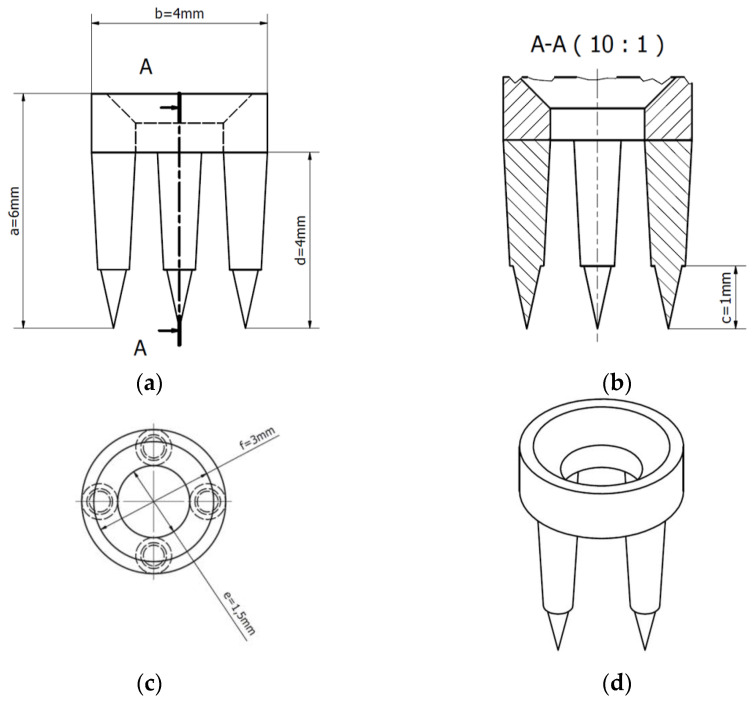
A technical drawing of the SD—(**a**) front view (elevation view); (**b**) sectional view (cut along A-A); (**c**) top view (plan view); (**d**) isometric view. Legend—a: ring height of 6 mm; b: ring diameter of 4 mm; c: maximum insertion size into the bone of 1 mm; d: leg height of 4 mm; e: internal diameter of 1.5 mm; f: external diameter of 3 mm.

The SD exhibits a symmetrical layout, as evident in the front view (Figure 3a) and top view (Figure 3c), highlighting the balanced distribution of its anchoring legs. This design improves load distribution and reduces localized stress concentrations in the bone.

The sectional view (Figure 3b) demonstrates the engineered tapering and spacing of the legs, which are optimized for insertion ease and enhanced stability once in position.

The isometric view (Figure 3d) provides a comprehensive visualization of the SD, showcasing the integration of all structural components to form a unified, high-precision device.

### 2.5. Simulation Parameters

A 10 N force was applied at a 30° angle to the vertical axis (Y) to simulate realistic orthodontic conditions. The force was transmitted from the mini-implant to the molar via the connector tube, effectively replicating the biomechanics of molar intrusion supported by skeletal anchorage. This angled application replicates the clinical load transfer during orthodontic procedures, providing a realistic analysis of stress and strain distribution in the periodontal ligament, alveolar bone, and adjacent tissues.

### 2.6. Validation and Reproducibility

To ensure reproducibility, all geometric modeling, material properties, and boundary conditions were consistently applied across simulations. The methodology was validated against previous studies, which demonstrated similar stress distributions and deformation patterns under comparable conditions. Additionally, all software settings and parameter inputs are available for replication upon request.

## 3. Results

### 3.1. Influence of SD on Mini-Implant Deformation: Comparative Analysis

#### 3.1.1. Equivalent von Mises Stresses

**Figure 4:**
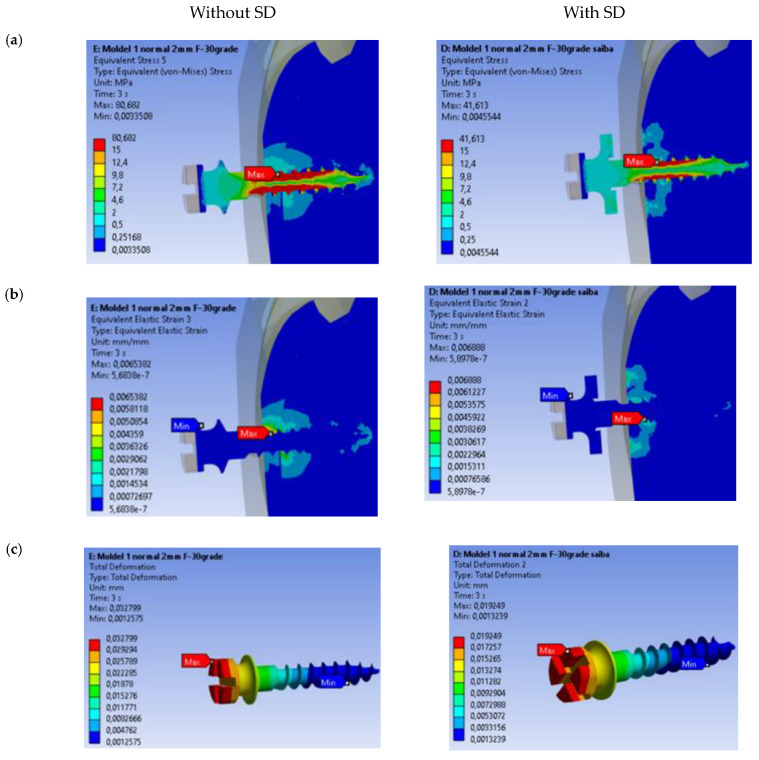
Comparative analysis of (**a**) equivalent von Mises stresses; (**b**) the state of linear equivalent specific deformations; (**c**) the total deformation state in the mini-implant, with and without the SD.

In Figure 4a, the stress state for the two analyzed models is presented. The maximum value obtained is 80.682 MPa for the case of the mini-implant without the SD. The minimum value obtained is 41.613 MPa for the case where the SD is present. From the results, it is observed that the maximum value in the presence of the SD is located at shallower depths. The volume of material solicited in the case of the anchored SD is much smaller in both the bone and mini-implant.

#### 3.1.2. Equivalent Deformations

In Figure 4b, the state of linear equivalent specific deformations for the two models is presented. The maximum value obtained is 0.006888 mm/mm for the model where the SD is present. From the results, it is observed that the aspect of the state of equivalent specific deformations is different for the two analyzed cases, with the maximum being located at different points at different depths. The volume of material solicited is much smaller in the case of the presence of the SD.

#### 3.1.3. Total Deformation of Mini-Implants

The analysis of total deformation in mini-implants under loading conditions is illustrated in Figure 4c, comparing the presence and absence of an SD. The maximum deformation value reaches 0.032799 mm, with notable stress concentrations at the interface of the implant and the surrounding material. This indicates that the absence of an SD results in increased displacement and reduced stability, likely due to the inability to evenly distribute mechanical forces across the system. The maximum deformation value is reduced to 0.019249 mm, demonstrating the role of SD stability in mitigating deformation. By introducing the SD, the mechanical forces are distributed more uniformly, significantly enhancing the structural rigidity of the system. The comparative results clearly highlight the functional advantage of incorporating an SD into the mini-implant design. The SD reduces peak deformation values, thereby minimizing potential stress-related failures and improving the overall mechanical stability of the implant under orthodontic loading conditions.

The findings from this analysis emphasize the critical importance of SD inclusion in applications requiring high stability and reduced deformation in biomechanical systems. Table 2 presents a comparative analysis of the biomechanical performance of mini-implants with and without the stabilization disc (SD) under orthodontic loading. It highlights key parameters such as von Mises stress, strain, and deformation.

**Figure 5:**
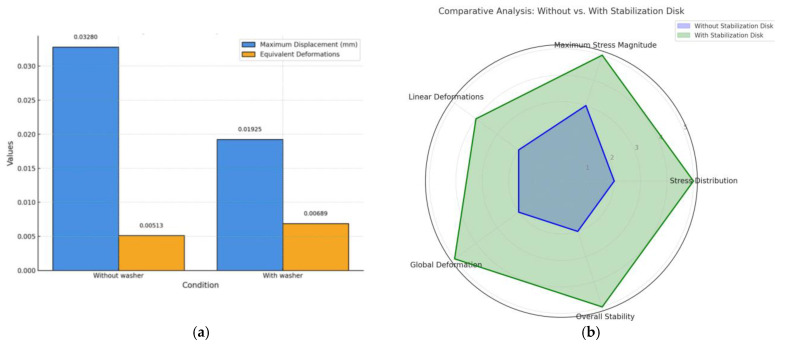
Influence of SD: (**a**) comparison of maximum displacement and equivalent deformations in mini-implants with and without SD; (**b**) spider chart comparison of mechanical performance in mini-implants with and without SD.

The SD shows (Figure 5a,b) a clear advantage across all analyzed parameters, including stress distribution, maximum stress magnitude, linear deformations, global deformation, and overall stability.

## 4. Discussion

The use of stabilization discs and similar modifications for enhancing mini-implant stability has garnered attention in orthodontic research. Studies indicate that various factors, including the design and dimensions of mini-implants, significantly influence their stability. For instance, Lee et al. emphasize that improving the shape, thread design, and surface treatment of mini-implants can enhance their stability under orthodontic loading conditions [20]. This assertion is supported by findings from Miglani and Cyan, who demonstrated that increasing the diameter of mini-implants leads to greater surface contact with the bone, thereby improving primary stability [21]. Moreover, Sahoo highlights that mini-implants exhibit superior bone-to-implant contact compared to standard implants, which is important for maintaining stability during orthodontic procedures [22]. This is further corroborated by Seifi and Matini, who found that wider and longer mini-implants enhance the mechanical interlock between the implant and bone, contributing to improved stability [23]. The importance of mechanical stability is echoed by Alsaeedi, who notes that primary stability is vital for the success of orthodontic mini-implants, as any loosening can lead to treatment failure [4]. In addition to design modifications, the insertion technique plays a critical role in achieving stability. Chandak et al. discuss how the primary stability of mini-implants is derived from the mechanical interlocking of the implant threads with the surrounding bone [24]. This mechanical interlocking can be optimized through careful consideration of the insertion angle and technique, as highlighted by Popa et al., who found that self-drilling mini-implants provide better initial stability due to increased bone contact [6]. Furthermore, the choice of materials also affects stability; Pan et al. noted that the conical shape of mini-implants can enhance primary stability, although excessive compression stress during insertion may lead to complications [25].

The clinical implications of these findings suggest that modifications such as stabilization discs could potentially enhance the mechanical interlock and surface area contact between the mini-implant and bone, thereby improving overall stability. This is particularly relevant in regions with variable bone density, where the design and insertion technique must be tailored to optimize stability [26].

The use of stabilization discs represents a flexible and innovative approach to enhancing the stability of orthodontic mini-implants without necessitating permanent modifications to their design or structure. Unlike adjustments to the shape, thread design, or surface treatment of mini-implants [20]—which require changes during the manufacturing process—a stabilization disc can be selectively applied by the clinician, depending on the specific requirements of the treatment or patient anatomy. Stabilization discs provide an additional mechanical interlock by increasing the surface area of contact between the implant and cortical bone. This improvement is particularly valuable in cases where bone quality or density may compromise the primary stability of a standard mini-implant. For instance, in areas of lower bone density, such as the posterior maxilla, the stabilization disc can distribute forces more evenly and reduce localized stress concentrations, as demonstrated in this study’s results through the reduction in von Mises stress values.

The clinical implications of reduced von Mises stresses on the longevity and stability of mini-implants are significant, particularly in the context of orthodontic applications. Von Mises stress is a critical factor in determining the mechanical stability of mini-implants, as it reflects the distribution of stress within the surrounding bone. Moreover, the stress distribution patterns vary with the dimensions of the mini-implants, suggesting that both length and diameter play important roles in managing the stress experienced during orthodontic procedures [1].

The implications of reduced von Mises stresses extend to the longevity of mini-implants. Lower stress levels are associated with decreased risk of bone necrosis and implant failure. Excessive stress can lead to microdamage and ischemia in the surrounding bone, which can delay healing and ultimately compromise implant stability [25]. For example, Pan et al. highlighted that excessive compression stress at the bone–implant interface could induce detrimental effects on the stability of mini-implants, emphasizing the importance of managing stress distribution [25]. Furthermore, Sarika et al. noted that increased stress concentrations could lead to mini-implant failure, reinforcing the need for optimal insertion angles to mitigate these risks [27].

As evident from Figure 4a and Figure 5a, the presence of the SD significantly reduces the maximum total displacements of the mini-implant by approximately 41.3% (from 0.03280 mm to 0.01925 mm). This reduction indicates that the SD enhances the stability of the screw during orthodontic treatment, as higher displacement values are observed in the absence of the SD. This outcome aligns with the physical principle that the SD increases the rigidity of the system.

From Figure 4b and Figure 5b, it is observed that the maximum von Mises stresses are reduced when the SD is employed. Without the SD, the maximum stress reaches 80.682 MPa, whereas, with the disk, it decreases to 41.613 MPa, resulting in a reduction of nearly 48.5%. Furthermore, the stress distribution is more uniform, and the volume of material subjected to high stress is smaller in both the mini-implant and the surrounding bone.

Several studies have quantified strain and deformation values for mini-implants, providing insights into their mechanical performance under various loading conditions. For instance, Sivamurthy and Sundari conducted a finite element analysis that revealed stress values for mini-implants with dimensions of 1.3 × 6 mm and 1.3 × 8 mm, showing a minimum stress of 19.85 MPa and a maximum of 43.34 MPa during retraction and intrusion, which are well within the fatigue limit of titanium [1]. This aligns with findings from Zhou et al., who noted that loading stress is primarily distributed in the cortical bone surrounding the implant neck, indicating that the design and placement of mini-implants significantly influence stress distribution and potential failure [8]. These findings suggest that the SD not only extends the lifespan of the mini-implant but also minimizes bone defects during orthodontic treatment. Additionally, in the cortical bone, maximum stress levels occur at greater depths in the presence of the SD. This is advantageous because cortical bone, due to its higher resistance compared to trabecular bone, tolerates higher stress levels, thereby contributing to the improved stability of the mini-implant during treatment. Regarding equivalent specific deformations (Figure 4c and Figure 5a), the SD reduces the deformation levels significantly. While the maximum deformation values are similar, the volume of material affected by deformation is much smaller with the SD. This demonstrates that the SD plays a vital role in minimizing deformations and distributing forces more evenly, thereby enhancing the overall stability of the mini-implant system.

The medical device (Figure 2 and Figure 3) developed for orthodontic anchorage is a flat SD with four prongs. The disc is crafted from robust and biocompatible materials, such as titanium or stainless steel, depending on the material composition of the orthodontic mini-implant. The SD has the following dimensions: (a) ring height, 6 mm; (b) ring diameter, 4 mm; (c) maximum insertion size into the bone, 1 mm; (d) leg height, 4 mm; (e) internal diameter, 1.5 mm; (f) external diameter, 3 mm. Each prong features a flat surface, designed to securely attach the SD to the bone and ensure its stable positioning throughout the orthodontic treatment. The device is placed between the mini-implant tip and the surrounding tissues (e.g., cancellous or cortical bone), where it serves as an additional support structure. The SD’s purpose is to bolster the stability and durability of mini-implants utilized for orthodontic anchorage. By inserting the interchangeable disk between the mini-implant and either the gingiva or bone, it enhances force distribution and decreases the likelihood of detachment or instability during orthodontic procedures. This ensures a more robust and reliable anchorage system, contributing to the efficacy and success of orthodontic treatments. The SD also addresses key challenges in orthodontic applications, such as ensuring secure anchorage and preventing mini-implant detachment. Acting as a securing component between the mini-implant and surrounding tissues, the interchangeable disk distributes applied forces evenly and alleviates pressure on the mini-implant insertion point within the bone. This reduces the risk of mobilization or loosening of the mini-implant during treatment.

This design minimizes the risk of mobilization or loosening during orthodontic procedures, ultimately contributing to more reliable and effective anchorage in orthodontic treatments. The impact of SDs on stress distribution in cortical and cancellous bone during orthodontic treatment is a critical area of research, particularly in the context of mini-implants used for anchorage. SDs’ role in modifying stress distribution can significantly influence the outcomes of orthodontic procedures, particularly regarding the stability of mini-implants and the health of surrounding bone structures.

Mini-implants, especially those placed in the infrazygomatic crest (IZC) and mandibular buccal shelf (MBS), have revolutionized orthodontic treatment by providing stable anchorage, minimizing root damage, and enabling precise three-dimensional control over tooth movement [28]. The ability to manipulate roll, pitch, and yaw simultaneously has significantly enhanced treatment outcomes, particularly in complex malocclusions where traditional methods fall short. However, the biomechanical potential of these systems is still limited by anatomical and physiological constraints [29,30]. The integration of stabilization discs (SDs) into mini-implant systems represents a promising innovation, addressing these challenges and improving clinical outcomes. Future studies focusing on long-term stability and refined biomechanical applications will be essential to further advance the role of mini-implants in modern orthodontics.

### Limitations of the Experiment

(a) Simplified model: The experimental setup relies on simplified geometric and biomechanical models, which may not accurately reflect the complexity of clinical conditions. For example, the interactions between various tissues, such as soft tissues and the bone interface, were not fully integrated. Future studies should incorporate more realistic models to improve the clinical relevance of the findings.
(b) In vitro conditions: The in vitro nature of this study limits its applicability to real-world clinical scenarios. Unlike in vivo conditions, where the biological environment introduces variability (e.g., bone remodeling, soft tissue dynamics, and patient-specific factors), the controlled laboratory setup lacks these dynamic interactions. This could lead to an overestimation or underestimation of the stabilization disc’s performance.
(c) Finite Element Analysis (FEA) assumptions: While FEA is a powerful tool for mechanical analysis, it is inherently limited by the assumptions and simplifications required to construct the models. For instance, isotropic and homogeneous material properties were assumed, which may not fully represent the anisotropic nature of bone or the complex interactions within the biomechanical system.
(d) Material properties: The material properties used in the simulations were idealized, assuming uniformity and consistency across all components. In clinical practice, variations in material properties due to manufacturing tolerances or aging effects could influence the performance of the mini-implant and stabilization disc.
(e) Single-variable analysis: This study primarily focused on the effects of the stabilization disc on mini-implant stability without addressing other influential factors, such as patient-specific variables (e.g., bone density, age, and health conditions) or alternative treatment protocols. A more comprehensive analysis incorporating these factors would provide a broader perspective on treatment outcomes.
(f) Short-term evaluation: The evaluation was limited to short-term mechanical performance under simulated conditions. Long-term effects, such as implant stability over months or years, and potential complications (e.g., bone resorption or implant failure) remain unexplored. These aspects are critical for understanding the full potential of the SD.
(g) Application in proximity to teeth and risk of soft tissue damage: While the stabilization disc provides enhanced anchorage stability, its application near teeth or areas with limited interproximal space could pose challenges. The proximity to adjacent teeth increases the risk of unintentional contact or interference during insertion, which may affect treatment outcomes. Furthermore, the presence of sharp prongs on the SD could increase the risk of soft tissue damage, particularly in regions with thinner gingival tissue. Future studies should evaluate the safety and efficacy of the SD in these specific anatomical contexts.
(h) Lack of in vivo validation and applicability to clinical practice: While this study offers valuable mechanical insights into the performance of the stabilization disc (SD) through finite element analysis (FEA), it is important to acknowledge the absence of in vivo validation as a limitation. Factors such as bone remodeling, tissue response to loading, osseointegration, and variations in patient anatomy are not accounted for in this study. These limitations may affect the direct applicability of the findings to real-world orthodontic practices, as clinical outcomes are influenced by dynamic factors.
(i) Bone adaptation: This study does not consider the biological remodeling and healing processes that occur in response to mini-implant placement and orthodontic loading. These processes may alter stress distributions over time.
(j) Force variability: The static forces applied in the simulation differ from the dynamic and variable forces experienced during actual orthodontic treatment.

Patient-specific factors, differences in bone density, cortical bone thickness, and individual variations in biomechanics are not included in the uniform model used.

## 5. Conclusions

The integration of stabilization discs into mini-implant systems represents a promising advancement in orthodontics, offering improved mechanical stability, enhanced stress distribution, and reduced deformation during treatment. By providing a flexible and adaptable solution, the stabilization disc addresses key challenges such as variable bone density and high orthodontic forces, without necessitating permanent modifications to implant design.

## Figures and Tables

**Table 1:** The model of material properties used in the finite element analysis [19].

Material/Component	Elastic Modulus (MPa)	Poisson’s Ratio
Cortical bone	17,000	0.3
Cancellous bone	350	0.25
Mini-implant	110,000/200,000	0.3
Bracket	380,000	0.19
Teeth	84,100	0.2
PDL	68.9	0.45

**Table 2:** Comparative analysis of stress, strain, and deformation with and without the stabilization disc (SD).

Condition	von Mises Stress (MPa)	Strain	Deformation (mm)
	Maximum	Minimum	Maximum
Without SD	88.662	0.0032508	0.056063
With SD	41.613	0.0035544	0.050877

## Data Availability

Data are contained within this article.
